# Health status and infections in patients with symptomatic primary and secondary immunoglobulin G (IgG) deficiencies receiving intravenous IgG replacement

**DOI:** 10.1186/s12865-020-00368-7

**Published:** 2020-06-29

**Authors:** Rudolf Weide, Roland Schnell, Christof Schardt, Michael Koenigsmann, Burkhard Otremba, Mark-Oliver Zahn, Jan Wierecky, Ute Braun, Manfred Hensel, Martine Klausmann, Doris Fleckenstein, Peter Ehscheidt, Stefan Feiten

**Affiliations:** 1 Praxis für Hämatologie und Onkologie Koblenz, Neversstr. 5, 56068 Koblenz, Germany; 2 pioh - Praxis Internistischer Onkologie und Hämatologie, Frechen, Germany; 3 Onkologische Gemeinschaftspraxis und Tagesklinik, Gelsenkirchen, Germany; 4 Onkologisches Ambulanzzentrum (OAZ), Hannover, Germany; 5 Onkologische Praxis Oldenburg / Delmenhorst, Oldenburg, Germany; 6 MVZ Onkologische Kooperation Harz, Goslar, Germany; 7 Überörtliche Gemeinschaftspraxis, Dres. Verpoort, Wierecky & Brandl, Schwerpunkt Onkologie & Hämatologie, Hamburg, Germany; 8 Gemeinschaftspraxis für Hämatologie und Onkologie, Ludwigshafen, Germany; 9 Mannheimer Onkologie Praxis, Mannheim, Germany; 10 Gemeinschaftspraxis Drs. Klausmann, Aschaffenburg, Germany; 11 MVZ Onkologie im Elisenhof, München, Germany; 12 Onkologische Praxis Dr. Ehscheidt, Neuwied, Germany; 13 Institut für Versorgungsforschung in der Onkologie, Koblenz, Germany

**Keywords:** Immunoglobulin G deficiencies, IgG replacement, Infections, Perceived health, Outpatient treatment

## Abstract

**Background:**

The effects of intravenous immunoglobulin G replacement on perceived health and infection susceptibility of patients suffering from immunoglobulin G (IgG) deficiencies should be evaluated in a prospective analysis.

**Methods:**

Patients with symptomatic primary or secondary IgG deficiencies were interviewed prior to the first IgG infusion (t_0_) and over the course of their treatment (t_1_ - t_6_). The respondents rated their current health using a 100 point scale (EQ-5D-5L), ranging from 0 (‘worst imaginable health’) to 100 (‘best imaginable health’). The patients also provided information on the frequency of infections and of infections requiring antibiotics in the past 8 weeks. A healthy control group (CG) without oncologic diseases answered the questions once.

**Results:**

One hundred six patients with a median age of 65 years (21–85 years) were investigated. The median serum IgG concentration changed from 500 mg/dl (t_0_) to 772 mg/dl (t_6_). The mean number of infections and of infections requiring antibiotics decreased during IgG replacement significantly. Current health according to EQ-5D-5L improved from 57 (t_0_) to 68 (t_6_), compared to 73 in the CG.

**Conclusion:**

During the course of IgG replacement patients reported fewer and less severe infections. Their health assessment improved but still was inferior to the healthy CG.

## Background

The administration of human polyvalent immunoglobulin G (IgG) is an effective way of preventing infections. Intravenous IgG (IVIG) replacement therapy has its first line indication in primary immunodeficiency disorders (PID) with reduced immunoglobulin production and increased susceptibility to infections due to genetic defects [1]. Secondary immunodeficiency disorders (SID) refer to hypogammaglobulinemic states due to B cell malignancies, prolonged immunosuppressive or cytostatic therapy, and hematopoietic stem cell transplantation (HSCT) associated with usually transiently reduced immunoglobulin production and increased susceptibility to infections. IVIG replacement therapy in secondary hypogammaglobulinemia has been shown to significantly reduce the infection rate in symptomatic SID and is widely used in patients with chronic lymphocytic leukemia (CLL), multiple myeloma (MM) and indolent non-Hodgkin’s lymphoma (NHL) [2–4].

The potentially life threatening risk of infections through bacteria such as *Streptococcus pneumoniae* and *Haemophilus influenzae* which are normally kept under control through antibody response, is clinically important particularly in the case of patients with immunodeficiencies. If the patients are found to suffer from such infections, therapy with antibiotics or IgG is indicated in order to prevent organ damage and death [1–4]. Randomized controlled studies have shown that IVIG replacement lowers the infection rate for CLL significantly [5–8]. A literature analysis that looked at randomized controlled studies showed that the risk of patients with lymphoproliferative diseases such as CLL and MM to develop interstitial pneumonia was reduced significantly when they were treated with polyvalent IgG and that clinically and microbiologically documented infections had decreased. There was however no proof of IgG replacement resulting in lower mortality rates [9].

The revised EMA (European Medical Agency) guideline on core SmPC (Summary of Product Characteristics) for human IVIG administration [4] provides a clear legal basis for IVIG replacement therapy in PID and SID.

Our report covers a prospective health status analysis of patients with symptomatic IgG deficiencies who were given IVIG at oncology group practices in Germany. We also looked at the benefits of IgG replacement therapy in order to reduce the number and severity of infections. A comparison of patients to a control group (CG) and a sub-analysis of patients with primary and secondary immune defects completed the analysis.

## Methods

Patients with symptomatic primary or secondary IgG deficiency who were about to start IVIG therapy were included. Overall, 12 sites took part in this multicenter study in Germany. Patients received IgG products supplied by different manufacturers. The IVIG dosage was chosen by the treating physician, there was no prespecified IVIG replacement protocol. Therefore the data reflect daily practice in routine care of IVIG replacement in community based oncology practices in Germany.

Interviews with the patients took place at the start of the treatment and were repeated at 8-weekly intervals during up to six measuring times. Treatment data and assessments of the oncologists in charge of treatment were linked with the data gained from the interviews.

In order to be better able to assess the results of the patients, a comparable non recurring survey was conducted in an age adjusted healthy CG with a similar sex distribution. ‘Healthy’ in this context meant that the respondents had no malignant or immunodeficiency disease and were therefore considered to be immune competent. The individuals of the CG were recruited with the help of a market research institute that carried out a Germany wide computer-assisted telephone survey.

Patients and CG assessed their current health status based on a validated 100 point scale (EQ-5D-5L) [10, 11] ranging from 0 (‘worst imaginable health’) to 100 (‘best imaginable health’). Only the EQ visual analogue scale (VAS) was used in order to record the patients’ self-rated health on a vertical VAS. Number and severity of infections were retrospectively assessed by the patients. An episode of infection was defined as any form of inflammation or infection and was purely self-reported by patients.

Forty-seven patients dropped out of the project over the course of the observation period due to different reasons; data of these patients were analyzed for all measurement times at which values were available. No patient was excluded from the analysis due to incomplete data.

Data was analyzed with the Statistical Package for the Social Sciences (SPSS) 19. Frequencies, medians, minima, maxima, means and standard deviations were calculated to describe the data. Data was analyzed for all patients and the two subgroups PID and SID. Patients suffering from MM were excluded from analyses of serum IgG level due to the disease related production of monoclonal immunoglobulin. Furthermore, IgA and IgM were not assessed for patients suffering from MM and Waldenstrom macroglobulinemia respectively.

T-tests for two independent samples were used to check the mean differences in age between patient and control group and between the patient subgroups for statistical significance.

The IgG values were transformed into ‘therapeutic’ (700 mg/dl or more) and ‘subtherapeutic’ (less than 700 mg/dl) categories; MM patients were excluded. The differences between the two groups (‘therapeutic’ vs. ‘subtherapeutic’) in the mean number of infections and of infections requiring antibiotics were checked for statistical significance over the entire observation period (t_0_ - t_6_) using independent samples t-tests. Based on the study design, the frequency of infections of the past 8 weeks was associated with the current IgG trough level.

Three repeated measures ANOVA (analyses of variance) were conducted to analyze the development of infections, infections requiring antibiotics and health status according to the EQ-5D-5L scale during the course of the treatment (t_0_ - t_6_). Single missing values did not exclude patients from these analyses. ANOVA were not analyzed regarding normal distribution since simulation studies have shown that ANOVA with repeated measurement are relatively robust against violations of the normal distribution assumption [12]. The Greenhouse–Geisser adjustment was used to correct if violations of sphericity occurred. Bonferroni adjusted post-hoc analyses were used to check the differences in mean values in the course of the treatment (t_0_ - t_6_).

All analyses were conducted by the Institute for Health Services Research in Oncology (Institut für Versorgungsforschung in der Onkologie), Koblenz.

## Results

### Study population

57 men (54%) and 49 women (46%) whose median age was 65 (21–85 years) could be interviewed at measuring time t_0_ and during the course of the treatment (t_1_ - t_6_). In the CG 55 men (55%) and 45 women (45%) with a median age of 66 (18–87 years) were examined. The mean age in both groups was 63 years (*p* = .659). Characteristics of patients and CG are shown in Table 1.

**Table 1:** Characteristics of patients and CG

	Patients (***n*** = 106)	CG (***n*** = 100)	
**Age**			
- median (range)	65 years (21–85)	66 years (18–87)	
- mean	63 years	63 years	*p* = .659
**Sex**			
- male	*n* = 57 (54%)	*n* = 55 (55%)	
- female	*n* = 49 (46%)	*n* = 45 (45%)	
**Indications for IgG replacement**			
- PID	*n* = 29 (27%)		
- SID	*n* = 77 (73%)		
**Underlying disease (only patients with SID)**			
- CLL	*n* = 27 (35%)		
- MM	*n* = 17 (22%)		
- follicular lymphoma	n = 5 (6%)		
- other NHL	*n* = 23 (30%)		
- other malignant diseases	n = 5 (6%)		

The majority of patients (73%) were given IgG due to SID, 27% due to PID. Patients with PID were statistically significantly younger than patients with SID (mean age 52 years vs. 66 years; *p* < .001). The sex distribution also differed. 72% of the patients with PID were female compared to 36% of the patients with SID.

Patients with SID received IgG due to CLL (35%), MM (22%), follicular lymphoma (6%), other NHL (30%) or other malignant diseases (6%). 58% of the patients with SID did not receive immunosuppressive treatment. The most frequently used immunosuppressive treatments provided to the remaining patients were rituximab (19%), corticosteroids (18%) as well as immunomodulatory drugs (lenalidomide and pomalidomide with 8%). In the 2 months preceding study entry, 95% of the patients had suffered from infections and 81% had suffered from infections requiring the use of antibiotics.

The median serum immunoglobulin values of all patients at time t_0_ amounted to 500 mg/dl for IgG (*n* = 89, without MM patients), 62 mg/dl for IgA (*n* = 87, without MM patients) and 26.5 mg/dl for IgM (*n* = 98, without patients suffering from Waldenstrom macroglobulinemia). Patients with PID had a median serum IgG of 600 mg/dl at t_0_ compared to 460 mg/dl in patients with SID (without MM patients).

The most frequently used IgG dosages were 30 g (40%), 10 g (38%) and 20 g (17%). The scheduled treatment intervals were 4 weeks in 80%, 3 weeks in 8% and 2 weeks in 6% of the cases. The mean IVIG dose infused per 4 weeks was 21 g (10 g - 70 g). Patients with PID had a mean dose of 19 g compared to 21 g in patients with SID.

### Changes in the course of the IgG replacement

#### IgG levels

**Fig. 1:**
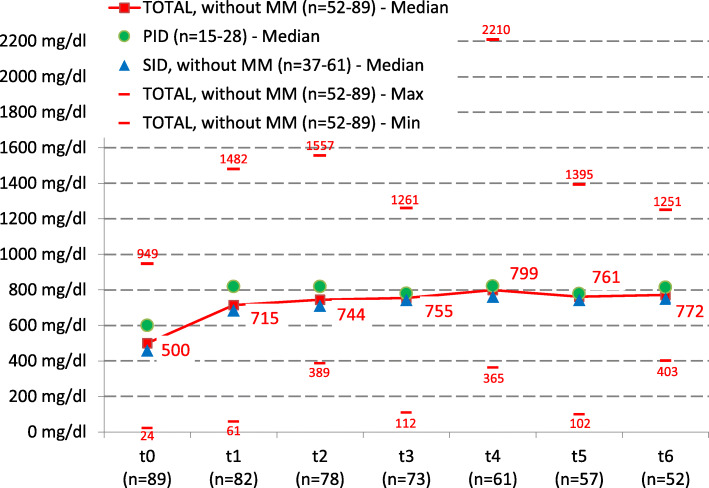
Development of IgG in the course of the replacement therapy (t_0_ - t_6_)

Under substitution, the median IgG value rose from 500 mg/dl (t_0_) to 772 mg/dl (t_6_) and was therefore at the lower end of the normal range. Patients with PID had an IgG of 600 mg/dl at t_0_ and of 815 mg/dl at t_6_. Patients suffering from SID (without MM patients) had a median IgG value of 460 mg/dl prior to the first IVIG administration and an IgG value of 751 mg/dl at t_6_. Figure 1 depicts the development of IgG in serum for the whole cohort and both subgroups.

#### Infections

Patients reported in a period of 8 weeks 1.8 infections (mean). This value fell continuously after the start of IVIG therapy, at t_6_ patients reported 0.7 infections in mean. A repeated measures ANOVA with a Greenhouse-Geisser correction determined that the mean number of infections showed a statistically significant difference between the measuring time points: F(4.4, 179.7) = 9.43, *p* < .001. Bonferroni adjusted post-hoc analyses revealed significant differences (*p* < .001) in the mean number of infections between t_0_ and t_2_ (1.07, 95%-confidence interval (CI)[0.48, 1.66]), t_0_ and t_3_ (1.12, 95%-CI[0.60, 1.64]), t_0_ and t_4_ (1.07, 95%-CI[0.53, 1.62]), t_0_ and t_5_ (1.12, 95%-CI[0.53, 1.71]) and t_0_ and t_6_ (0.98, 95%-CI[0.24, 1.71]). A mean difference in infections of 0.67 between t_0_ and t_1_ was not statistically significant. All post-hoc comparisons between measurement times t_1_ - t_6_, i.e. after the start of IgG administration, were not significant.

#### Infections requiring antibiotics

In a period of 8 weeks patients reported a mean number of 1.3 infections requiring the use of antibiotics. The number of infections requiring antibiotics fell continuously after the start of IgG replacement therapy, too. At t_6_ 0.3 infections requiring antibiotic treatment per patient were reported. A repeated measures ANOVA revealed statistical significance between the measuring time points: F(6, 198) = 9.64, *p* < .001. Bonferroni adjusted post-hoc analyses revealed significant differences in the mean number of infections requiring antibiotics between t_0_ and t_1_ (0.65, *p* = .002, 95%-CI[0.08, 1.22]), t_0_ and t_2_ (0.77, *p* = .002, 95%-CI[0.19, 1.34]), t_0_ and t_3_ (0.85, *p* < .001, 95%-CI[0.33, 1.38]), t_0_ and t_4_ (0.94, *p* < .001, 95%-CI[0.39, 1.50]), t_0_ and t_5_ (1.00, *p* < .001, 95%-CI[0.39, 1.61]) and t_0_ and t_6_ (0.94, *p* < .001, 95%-CI[0.35, 1.53]). The same pattern can be seen for infections requiring antibiotics as for infections in general: all post hoc comparisons between measurement times t_1_ - t_6_, i.e. after the start of IgG replacement, failed to reach statistical significance.

#### Infections and infections requiring antibiotics - subgroups

Patients with PID reported more infections than patients with SID. In both groups a substantial decrease in the number of infections could be observed after initiation of IVIG therapy.

Patients with PID had 2.3 infections in an 8 weeks time period. The mean values developed as follows: 1.2 (t_1_), 1.6 (t_2_), 0.9 (t_3_), 0.8 (t_4_), 1.2 (t_5_) and 1.1 (t_6_). Patients with SID had a mean number of 1.6 infections prior to the first IVIG administration. The mean values developed as follows: 1.0 (t_1_), 0.8 (t_2_), 0.6 (t_3_), 0.6 (t_4_), 0.4 (t_5_) and 0.6 (t_6_).

A similar pattern can be seen for infections requiring antibiotics. Patients with PID had more infections requiring antibiotics and in both groups the number of infections decreased. Patients with PID had 1.4 infections requiring antibiotics in an 8 weeks time period. The mean values developed as follows: 0.6 (t_1_), 0.6 (t_2_), 0.5 (t_3_), 0.4 (t_4_), 0.5 (t_5_) and 0.3 (t_6_).

Patients with SID had a mean number of 1.2 infections requiring antibiotics. The mean values developed as follows: 0.7 (t_1_), 0.5 (t_2_), 0.4 (t_3_), 0.3 (t_4_), 0.2 (t_5_) and 0.3 (t_6_).

#### Infections and infections requiring antibiotics – comparison between patients and CG

As far as the number of infections or infections requiring the use of antibiotics is concerned, there were substantial differences between CG and patients prior to the start of IgG replacement (t_0_) and during the course of IVIG treatment (t_1_ - t_6_). The mean number of infections was 1.8 (t_0_) and 0.7 (t_6_) in patients versus 0.2 in the CG. The number of infections requiring the use of antibiotics decreased during the course of the replacement therapy in the patient group (1.3 (t_0_) and 0.3(t_6_)) but remained above the level of 0.1 in the CG.

Descriptive statistics of infections and of infections requiring antibiotics are shown in Table 2.

**Table 2:** Descriptive statistics of infections and of infections requiring antibiotics

	Infections				Infections requiring antibiotics			
	Mean	Standard deviation	Min	Max	Mean	Standarddeviation	Min	Max
**Patients t0 (*n*** **= 106)**	1.8	1.0	0	5	1.3	0.9	0	5
**Patients t1 (*n*** **= 101)**	1.1	1.2	0	6	0.7	0.9	0	4
**Patients t2 (*n*** **= 95)**	1.0	1.5	0	8	0.5	0.9	0	4
**Patients t3 (*n*** **= 84)**	0.7	0.9	0	3	0.4	0.8	0	3
**Patients t4 (*n*** **= 72)**	0.7	0.6	0	2	0.3	0.6	0	2
**Patients t5 (*n*** **= 70)**	0.7	0.8	0	4	0.3	0.6	0	3
**Patients t6 (*n*** **= 59)**	0.7	1.0	0	4	0.3	0.6	0	2
**CG (*n*** **= 100)**	0.2	0.4	0	2	0.1	0.3	0	1

**Fig. 2:**
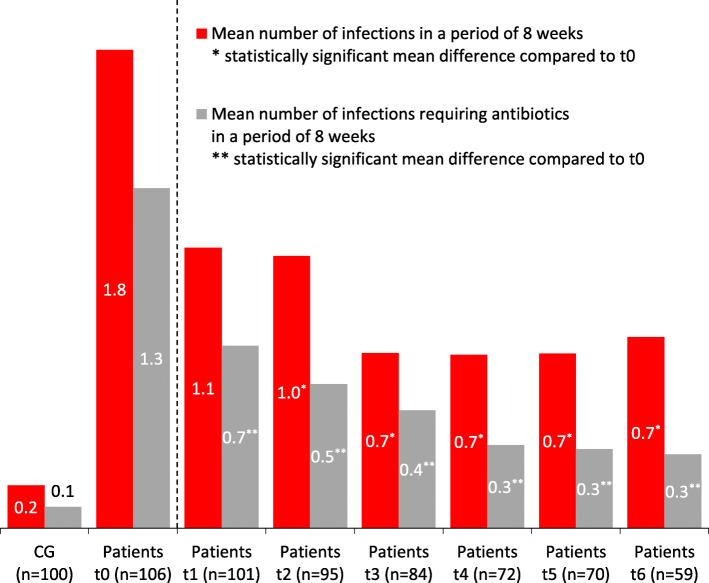
Frequencies of infections and of infections requiring antibiotics (mean values) in the course of the replacement therapy (t_0_ - t_6_)

Figure 2 depicts the decrease of infections and of infections requiring antibiotics after IgG replacement.

#### Infections and infections requiring antibiotics – effects of IgG concentration

Significant differences between ‘therapeutic’ (700 mg/dl or more) and ‘subtherapeutic’ (less than 700 mg/dl) IgG concentrations with regard to the number of infections could be observed. Over the entire observation period a mean of 0.8 infections was found in the ‘therapeutic’ group compared to 1.2 infections in the ‘subtherapeutic’ (*p* = .001).

Furthermore, a mean of 0.4 infections requiring antibiotics were reported in the ‘therapeutic’ IgG concentration group compared to 0.9 in the ‘subtherapeutic’ (*p* < .001).

#### Perceived health

Patients’ perceived health according to the EQ-5D-5L scale improved continuously in the course of the replacement therapy. At t_0_ a value of 57 was observed, at t_6_ of 68. A repeated measures ANOVA with a Greenhouse-Geisser correction showed a statistically significant difference between the measuring time points: F(4.4, 240.3) = 7.95, *p* < .001. Bonferroni adjusted post-hoc analyses revealed significant differences in the mean health values between t_0_ and t_1_ (9.64, *p* = .003, 95%-CI[2.17, 17.12]), t_0_ and t_2_ (12.61, *p* < .001, 95%-CI[4.47, 20.74]), t_0_ and t_4_ (9.34, *p* = .015, 95%-CI[1.05, 17.63]), t_0_ and t_5_ (11.93, *p* < .001, 95%-CI[4.10, 19.76]) and t_0_ and t_6_ (10.86, *p* = .006, 95%-CI[1.90, 19.82]). A mean difference of 8.95 between t_0_ and t_3_ was not statistically significant. Further post-hoc analyses between the measurement times t_1_ - t_6_, i.e. after the start of the IgG replacement, were not statistically significant with regard to perceived health.

Patients with PID and SID had quite comparable values in their health assessments. The biggest difference of 4 points could be observed at t_0_. In a methodologically not quite unproblematic comparison of the subjectively assessed health, patients (68, t_6_) did not completely reach the level of the CG (73).

**Fig. 3:**
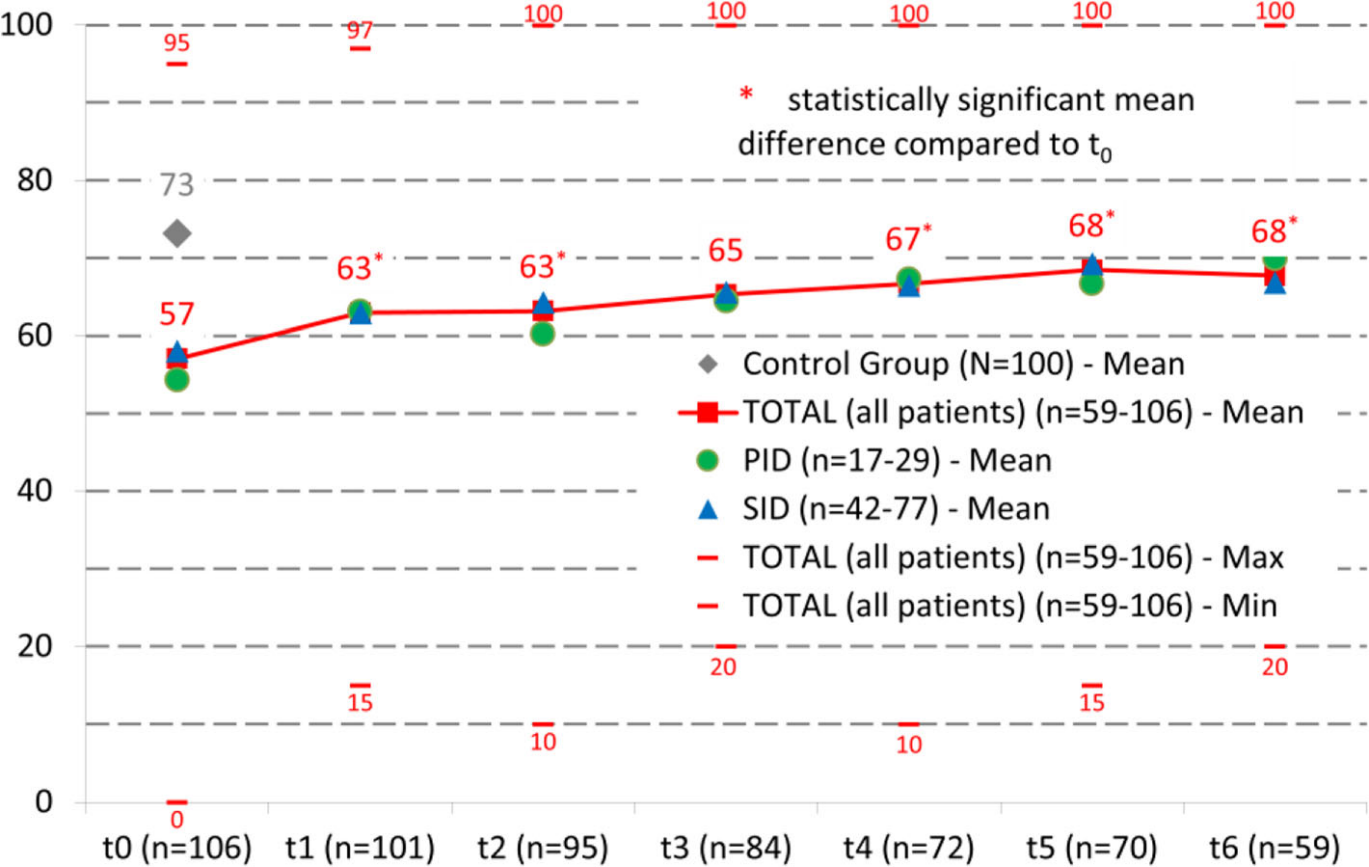
Health assessment (mean values) in the course of the replacement therapy (t_0_ - t_6_)

Figure 3 depicts the development of patients’ health status during the course of the IgG replacement, compared to a single value for the CG.

### Assessments of the treating oncologists

35% of the replacement therapies ended prior to the last planned interview (t_6_): in 13% the patient was no longer available, in 7% the deficiency was no longer in need of treatment, in 6% the patients refused further IgG replacement and in 2% it was terminated due to intolerances. In 8% the reason for the premature termination could not be determined. According to the conclusive assessment of the treating oncologists (t_6_) 94% of the patients treated had benefitted from the therapy (64% ‘greatly’, 30% ‘a little’). Retrospectively, the treatment was considered ‘very important’ (50%) or ‘important’ (44%) for the patient and continuation appeared ‘useful’ or ‘very useful’ in 86% of the cases. In most cases, oncologists could not think of ways of optimizing the therapy (95%).

## Discussion

### Reduction of infections

Among the examined patient population with primary and secondary antibody deficiencies, IgG replacement led to a decrease in the number and severity of infections accompanied by a simultaneous increase in the median IgG concentration from 500 mg/dl before the first IVIG administration to 772 mg/dl after the last infusion. Hence, the value was within the normal range albeit at the lower end. The number of patients without infections requiring the use of antibiotics rose from 19% at time t_0_ (*n* = 106) to 80% at time t_6_ (*n* = 69). Overall, a statistically significant decrease in the frequency of infections and of infections requiring antibiotics could be observed at the earliest follow-up appointment after the start of IVIG therapy and the numbers remained on a reduced level over the course of the therapy after this significant reduction. This data concurs with other studies that also showed a reduction of infections among patients treated with IgG. Hence, according to an observational study [13] involving patients with SID who were treated with IVIG, the number of bacterial infections could be reduced from 2.4 per patient in the 3 months prior to the start of IgG replacement to 0.7 per patient per year during IVIG.

According to actual EMA guidelines an IgG therapy is not indicated until the patient shows a significant antibody deficiency and/or shows proven specific antibody failure and suffers, in addition, from recurrent severe infections refractory to antibiotic therapy [4]. Furthermore, antibiotic therapy options must have been exhausted or are not indicated [4]. Concerning etiologies in the use of IVIG replacement therapy the EMA guidelines have changed from few etiologies (CLL, MM, HSCT, pediatric AIDS) to broader replacement criteria: SID in patients who suffer from severe or recurrent infections, ineffective antimicrobial treatment and either proven specific antibody failure or serum IgG level of less than 4 g/l [4]. With this new rule physicians are now allowed to also implement IVIG replacement therapy in an increasing number of SID cases following immunosuppressive therapy in systemic autoimmune diseases and intensive chemotherapy in solid organ transplantation.

In medical practice, we often see that most symptomatic patients have more than 2 infections per year needing antibiotic therapy. Based on our experience, a significant percentage of symptomatic patients with indolent lymphomas benefits from a targeted serum level adjusted IgG therapy. A clear reduction of infections requiring the use of antibiotics and an improvement of the patients’ quality of life serve as proof [14].

The normal therapy schedule consists of a four-weekly administrations of 30 g of IVIG. The aim is to achieve trough serum levels of 700 to 1000 mg/dl. Based on our experience, higher IgG levels are not necessary as they would not offer the patients a measurable clinical benefit. Previous analyses, too, have shown that IgG levels between 700 and 1000 mg/dl are sufficient to reduce the frequency of severe infections. On the other hand, we were able to show that a ‘subtherapeutic’ IgG concentration of less than 700 mg/dl resulted in a lower reduction of infections and, in particular, of infections requiring antibiotics. In this analysis, a median serum level of 772 mg/dl, i.e. a value at the lower end of the normal range was achieved, which may be due to the fact that more than half of the patients had only been given 10 g (38%) and/or 20 g (17%) of IgG per treatment cycle. The results presented in this context reflect the everyday experience in community based oncology group practices in Germany. When comparing our data with that of other analyses, the question remains as to whether a dose of 30 g (or 0.4 g/kg body weight) for patients with repeated infections could possibly have led to a better result [5, 7, 13, 14]. Recently a cohort study of 8633 patients from a tertiary referral center receiving rituximab has been presented. Rituximab led to an increase in severe infections. 85% had no measurement of immunoglobulin levels before rituximab therapy. Only 4.5% received immunoglobulin replacement therapy. Higher cumulative replacement dose was associated with a reduced risk of severe infections [15].

### Health improvement

Based on the EQ-5D-5L, the patients’ health improved on average from 57 (t_0_) to 68 (t_6_). 64% of the doctors in charge of treatment claimed that their patients had benefitted ‘greatly’ from the IVIG treatment whilst 30% said that they had benefitted ‘a little’ and according to 6% patients had benefitted ‘not at all’. At time t_6_ 65% of the patients had continued with the IgG replacement while 35% had stopped it, due to a variety of reasons (patient not available anymore, antibody deficiency no longer required treatment, patient rejected further IgG replacement, side effects).

As far as we know there is, up to now, very little published data available about the perceived health of patients receiving IVIG. This prospective analysis confirms the results of a prospective survey published in 2015 concerning patients with indolent NHL and symptomatic antibody deficiency [14]. It also showed that the rise of serum IgG levels led to fewer infections requiring the use of antibiotics [14].

When comparing the perceived health between patients and CG we observed an improvement in the patients from 57 (t_0_) to 68 (t_6_) during IVIG treatment, but it was still inferior to the healthy CG who scored 73 on the EQ-5D-5L. This shows that, despite IVIG treatment, patients’ health remains inferior to persons without a malignant disease, which is multifactorial and probably partly caused by chronic fatigue. Large cohort studies have shown that patients with common variable immune deficiency (CVID) and long term survivors of malignant lymphomas suffer from increased chronic fatigue [16, 17].

### Methodological considerations

The present study is a prospective multicenter patient survey. In addition, medical data, such as serum IgG level were transferred from the treatment files and the treating oncologists assessed the success of treatment with the last IgG administration. The data were linked and processed and are probably representative of the treatment reality of patients in hematological and oncological group practices in Germany. When carrying out the project, however, methodological compromises had to be made. Methodological limitations result from the rather small population of 106 patients. There was also no a priori calculation of the sample size. From a practical point of view, however, it must be noted that these patients are rare and that they had to be interviewed before the first IgG administration. A comparison of the patient subgroups with primary vs. secondary immunodeficiencies was therefore only possible on a descriptive level. The patient group may not have been homogeneous enough, even if the results found seem to apply to both subgroups.

Another methodological limitation is that a central parameter of this analysis, namely the number of infections and the number of infections requiring antibiotics, is based solely on the patients’ self-assessments. However, since we did not only want to capture infections requiring treatment, patients’ assessments was the only possible data source.

Although the patients formed their own control group through the repeated measurement, we wanted to assess the results using a ‘gold standard’ consisting of healthy, comparable people. For practical and financial reasons, this group could only be interviewed once. A comparison with the results of this CG is therefore of limited value. Moreover, interpreting the data of the CG, must take into account that the healthy probands were interviewed in August and therefore may have reported less infections and less infections requiring antibiotics.

Perhaps the biggest methodological problem is the comparatively high dropout rate of 35%, i.e. 35% of all patients did not provide data at all times. However, the effects found seem to be so robust that despite the dropouts they are still significant.

## Conclusion

In conclusion, patients with a symptomatic IgG deficiency had a median IgG value of 500 mg/dl (without MM patients) prior to the first IgG administration. In the course of the replacement therapy that value rose continuously to more than 700 mg/dl, reaching the lower end of the normal range. Both the number of infections and the number of infections requiring the use of antibiotics decreased continuously with an increasing IgG concentration. Patients’ perceived health improved accordingly. IgG dosage and IgG concentration respectively were associated with frequency and severity of infections.

## Data Availability

The datasets used and analyzed during the current study are available from the corresponding author on reasonable request.

## Abbreviations

IgG: Immunoglobulin G
CG: Control Group
IVIG: Intravenous Immunoglobulin G
PID: Primary Immunodeficiency Disorders
SID: Secondary Immunodeficiency Disorders
HSCT: Hematopoietic Stem Cell Transplantation
CLL: Chronic Lymphocytic Leukemia
MM: Multiple Myeloma
NHL: Non-Hodgkin’s Lymphoma
EMA: European Medical Agency
SmPC: Summary of Product Characteristics
VAS: Visual Analogue Scale
SPSS: Statistical Package for the Social Sciences
ANOVA: Analyses of Variance
CI: Confidence Interval
AIDS: Acquired Immune Deficiency Syndrome
CVID: Common Variable Immune Deficiency
